# Effectiveness of a type 2 diabetes prevention program combining FINDRISC scoring and telephone-based coaching in the French population of bakery/pastry employees

**DOI:** 10.1038/s41430-019-0472-3

**Published:** 2019-07-17

**Authors:** Philip Böhme, Amandine Luc, Pascal Gillet, Nathalie Thilly

**Affiliations:** 1 CHRU de Nancy, Service d’Endocrinologie, Diabétologie, Nutrition, F-54511 Vandœuvre-Lès-Nancy, France; 2 Université de Lorraine, EA 4360 APEMAC, F-54000 Nancy, France; 3 CHRU Nancy, Plateforme d’Aide à la Recherche Clinique, F-54511 Vandœuvre-Lès-Nancy, France; 4 MEDIALANE, Plateforme de télésanté, F-54320 Maxéville, France

**Keywords:** Risk factors, Epidemiology

## Abstract

**Background/objectives:**

Preventive actions targeting the risk of type 2 diabetes mellitus (T2D) and deployed from the workplace are scarce. This study aimed to measure this T2D risk in a large sample of the bakery/pastry employees in France and to assess the effectiveness of a telephone coaching program in participants with the highest risk.

**Subjects/methods:**

A screening survey using the FINDRISC score was conducted by phone among the employees. Those with a moderate risk (score ≥ 12 and <15; body mass index ≥ 25 kg/m^2^) or high/very high risk (score ≥ 15) were invited to participate in a 6-month coaching program including 6 monthly interviews together with a final evaluation interview three months later. The effects and impact were evaluated using 8 questions on dietary knowledge/behavior as well as the GPAQ (physical activity) and SF-12 (quality of life) questionnaires.

**Results:**

There were 19,951 employees eligible for screening (age: 38.0 ± 13.5 years, men 49.6%, mean FINDRISC score 5.9 ± 4.4). A high/very high score was found in 4% of individuals. Overall, 1,348 (among 2,018) eligible employees agreed to participate in the coaching program, 630 of whom participated in all interviews. Of the latter, dietary knowledge/behavior (+1.60) and quality of life (+1.83) improved (*P* < 0.0001), with a favorable trend for physical activity (+0.06, *P* = 0.0756). Dietary knowledge/behavior continued to improve in the 581 completers (+0.17, *P* = 0.0001).

**Conclusions:**

This two-step prevention program associating T2D risk estimation and a 6-month telephone coaching was deployed in the French craft bakery/pastry sector with significant adhesion. Such program appears beneficial for enhancing knowledge and mobilizing skills associated with T2D prevention.

## Introduction

Diabetes mellitus now affects 415 million people worldwide, 91% of whom have type 2 diabetes mellitus [1]. France has not been spared by this increase, with 3.3 million individuals diagnosed with diabetes in 2015 incurring high public health costs [2]. Type 2 diabetes is a common chronic disease in both the general and working population, resulting in an increased incidence of absenteeism at work, and increased morbidity and mortality [3, 4]. The rapid increasing prevalence of the disease is largely linked to lifestyle factors associated with overweight, including changes in dietary habits and increasing sedentary behavior [5]. Accordingly, people with significant risk for type 2 diabetes would benefit from early identification and lifestyle intervention [6, 7]. Many authors have proposed diabetes risk scores that can be easily applied in community settings [8–10]. Among these, the Finnish Diabetes Risk Score (FINDRISC) is intended for use directly by non-specialists and recommended by several guidelines [11–15]. Controlled lifestyle interventions can delay the development of type 2 diabetes in high-risk populations although translating these findings into real-world primary healthcare practice or professional environment remains difficult [16–18]. Health coaching is recognized as an innovative strategy to mobilize positive health behaviors and probably facilitates prevention actions [19]. In particular, telephone counseling support allows delivering lifestyle interventions in a broad and personalized manner [20].

The aim of this study was first to evaluate the risk of type 2 diabetes in bakery/pastry employees working in France using the FINDRISC score and to subsequently evaluate the effectiveness of a lifestyle intervention by telephone coaching, proposed to the screened individuals with a significant risk for type 2 diabetes.

## Methods

A cross-sectional survey was first conducted to assess the risk of developing type 2 diabetes in bakery/pastry employees working in France. This survey was carried out by Medialane®, at the request of the health insurance company in charge of prevention actions for all bakers/pastry employees in France (AG2R-La Mondiale). Medialane® is a multi-service company specialized in telehealth activities, in particular, health-related surveys or coaching programs by phone.

**Fig. 1:**
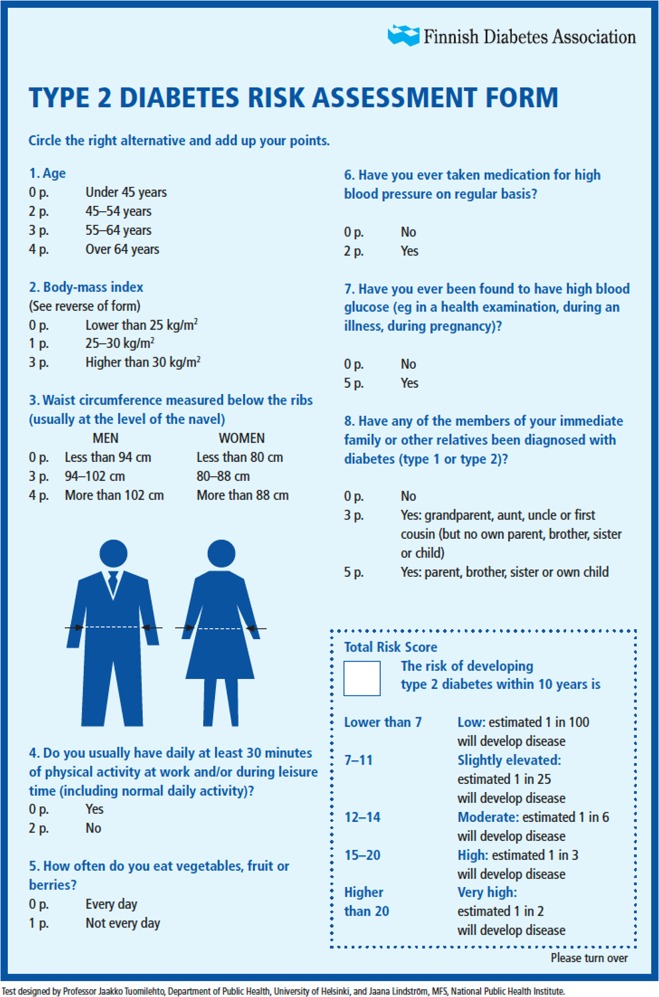
The FINDRISC questionnaire (as currently available at https://www.diabetes.fi/files/502/eRiskitestilomake.pdf; accessed 6 December 2018)

The database of all employees working in the French bakery/pastry sector (*n* = 96,039) aged ≥ 16 years, with their names, personal and professional addresses and telephone numbers, was obtained from their health insurance company (exhaustiveness >90%). Employees were contacted by phone by six nurses from Medialane® between May 2015 and May 2016 in random order. Two questions allowed identifying ineligible individuals (i.e., individuals with current diabetes, retired individuals or those who had changed profession) who were subsequently excluded from the screening study. If eligible, employees were asked to participate in a survey to evaluate their 10-year risk of type 2 diabetes. The telephone contacts were discontinued when the inclusion goal of 20,000 individuals was reached. This number was defined from the Medialane nurse resources to conduct the survey and in order to obtain a sufficient precision in the estimation of the mean FINDRISC score (± 0.07, considering a standard deviation of 5 according to previous studies) [21]. The FINDRISC questionnaire used for this survey was developed in 2001 from a cohort study of a representative random sample of the Finnish adult population in order to predict the 10-year incidence of drug-treated type 2 diabetes [9]. It has since been tested in various countries [12, 14, 22, 23]. This questionnaire includes eight items and each self-reported answer is weighted according to the risk increase with a final score ranging from 0 to 26 (Fig. 1). A FINDRISC score lower than 7 is linked to a very low type 2 diabetes risk, 7–11 to a low risk, 12–14 to a moderate risk, 15–20 to a high risk, and 21–26 to a very high risk, with these five categories corresponding to a probability of developing type 2 diabetes within the next 10 years of 1%, 4%, 17%, 33%, and 50%, respectively. Anthropometric data (height, weight, waist circumference measured in a horizontal plane, midway between the inferior margin of the ribs, and the superior border of the iliac crest) were self-measured by respondents, with body mass index (BMI) calculated by the Medialane® staff. The Medialane® nurses also collected additional data including gender, type of work (selling vs. production) and whether they worked full- or part-time. The mean duration of this first phone interview was 17 min.

At the end of the screening phase, a coaching program has been proposed to employees aged 16–60 years, who presented either a moderate risk (12 ≤ FINDRISC score ≤ 14) and overweight or obesity (BMI ≥ 25 kg/m^2^), or a high or very high risk (FINDRISC score ≥ 15) regardless of their BMI. The coaching program was conducted between June 2015 and March 2017 by the same Medialane® nurse team who performed the screening, all of whom were trained in motivational interviewing. The program consisted of six-monthly telephone interviews lasting ~20 minutes, the first and the last (sixth) of which included a short evaluation time of ~5 min using various tools mentioned hereafter. Throughout these interviews, the nurses assisted the individuals through collaborative conversations with the following incentive messages: more healthy eating, increased daily physical activity, weight management, and stress reduction. A final and seventh telephone interview was scheduled 3 months after the end of the last intervention coaching interview, in order to assess the maintenance of the benefit of the program. Physical activity was estimated by the GPAQ (Global Physical Activity Questionnaire). The GPAQ covers several components of physical activity, such as intensity, duration, and frequency, and it assesses three domains in which physical activity is performed (occupational physical activity, transport-related physical activity, and physical activity during discretionary or leisure time). Finally, it allows classification of the individuals into one of the three following categories linked to activity intensity: 1 (limited), 2 (medium), or 3 (high activity). The English and French versions of this questionnaire are available on the WHO website at https://www.who.int/ncds/surveillance/steps/GPAQ_EN.pdf and https://www.who.int/ncds/surveillance/steps/GPAQ_FR.pdf, respectively.

Health-related quality of life was measured using the Short-Form 12 (SF-12), whose final score ranges from 17 (worst quality of life) to 55 (best quality of life) [24, 25]. Dietary knowledge and behavior were assessed by a questionnaire built by the research team (Table 1): each correct answer counting for one point toward the total score. The level of motivation or commitment, inspired by the Prochaska and Di Clemente scheme, was also assessed together by the coaching nurse and the participant, by using only four categories: pre-contemplation, contemplation, preparation, and action [26].

**Table 1:** Eating knowledge and behavior questionnaire

Categories	Question or assertion	Scoring
Knowledge questions	Starchy foods make you fat	Wrong = 1 Right = 0
	Fish contains less protein than meat	Wrong = 1 Right = 0
	There is salt in breakfast cereals	Wrong = 0 Right = 1
	Frozen products contain fewer vitamins than fresh products	Wrong = 1 Right = 0
Behavior questions	Regardless of their form (raw, cooked, plain or prepared), you should eat at least 5 fruits and vegetables per day	No = 0 Yes = 1
	You should eat starchy foods (bread, cereals, potatoes, etc.) at each meal	No = 0 Yes = 1
	You should consume fish at least twice a week	No = 0 Yes = 1
	You should systematically salt your food before tasting it and you systematically re-salt your dishes	No = 1 Yes = 0
Minimum–maximum score		0 to 8

The main outcome for the screening phase was the FINDRISC score, considered as a continuous and categorical variable (very low, low, moderate, high, or very high risk). FINDRISC scores were calculated overall, as well as in respondents ≥ 30 years old, >45 years old, and respondents >45 years having at least one of the main risk factors for diabetes collected for this survey (BMI ≥ 28 kg/m^2^, history of hypertension, or a first degree relative with diabetes) as proposed earlier by the French recommendations [27]. Participation to part or the entire program was also described. Continuous variables are presented as means and standard deviations and categorical variables as numbers and percentages. Three outcomes of interest were used to evaluate the effectiveness of the coaching program: variations in physical activities (GPAQ), in dietary knowledge and behaviors, and in quality of life (SF-12). As these three variables do not follow a normal distribution, results are presented as medians and interquartile ranges. The evaluation of the effects of the coaching program consisted in the comparison of the questionnaire results between the first and the sixth interview, whereas the evaluation of its impact consisted in the comparison between the sixth and the last (seventh) interview (three months after the last coaching interview). A modified intention to treat analysis (mITT) was performed on all individuals with a high FINDRISC score who agreed to participate (whether they were eligible or not) and attended to all telephone interviews [28]. A per-protocol analysis (PP) was focused on the employees eligible for coaching and attended to all interviews. Analyses comparing the scores between interviews one and six (or six and seven) was performed using Wilcoxon signed rank test on paired samples. A symmetry test (equivalent to the McNemar’s test but for more than two response modalities) was used on matched nominative data to determine changes in motivation’s stages between the first and the last interview. A *P* value of <0.05 for two-sided tests was considered significant. All analyses were performed with SAS version 9.4 (SAS Institute, Inc., Cary, NC, USA).

The survey was conducted according to the principles of the Declaration of Helsinki and approved by a national Ethics Committee (*Commission Nationale de l’Informatique et des Libertés*). All employees contacted by phone were informed regarding the survey protocol and that their participation in the survey and telephone coaching was voluntary, anonymous and without any compensation.

## Results

**Fig. 2:**
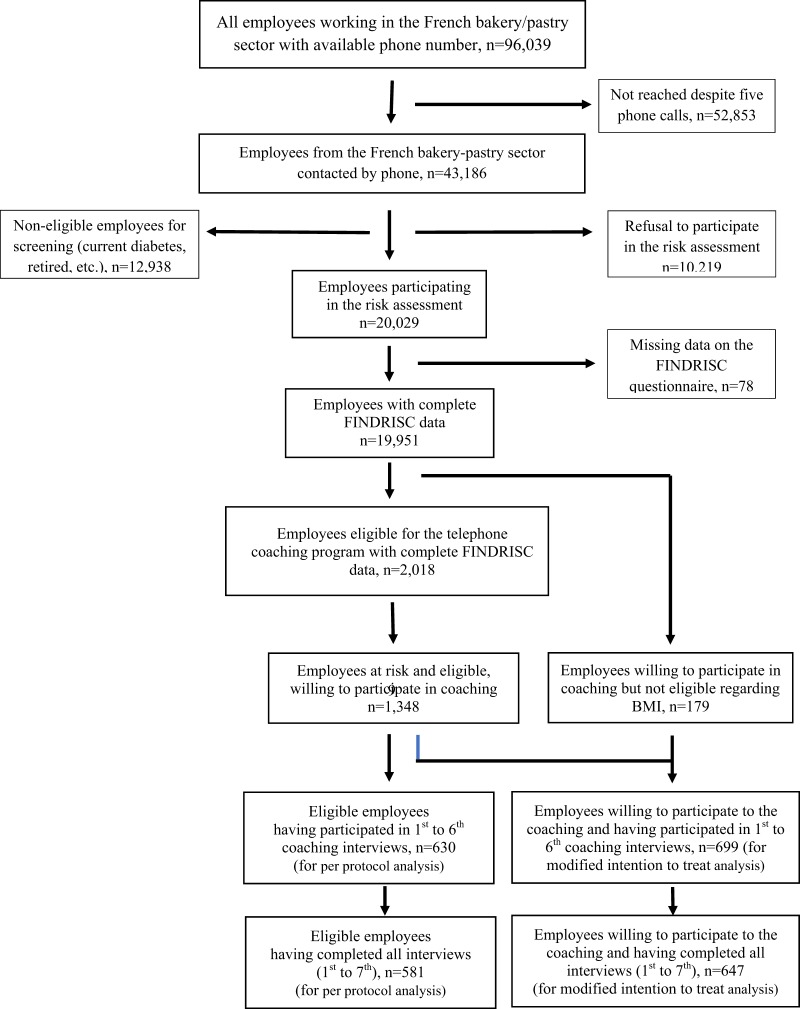
Study flowchart (including screening and intervention phases)

Among the 96,039 employees working in the French craft bakery/pastry sector, 30,248 eligible individuals were contacted by phone and asked to participate in the screening survey (Fig. 2). Among these, 20,029 agreed to participate (66.2%). The main reason for refusal (*n* = 10,219) was a lack of time to answer questions. The presented results for the screening phase stem from the 19,951 (99.6%) respondents with no missing data. Among these, 2018 were high-risk individuals eligible for the coaching program. Of the 1, 521 individuals who agreed to participate in this program, 179 did not meet the inclusion criteria since, despite a FINDRISC score between 12 and 14, but BMI <25 kg/m^2^). Finally, 1,348 (66.8%) eligible individuals accepted to participate and 581 (28.8%) participated in all scheduled interviews (including the seventh).

The characteristics and responses to the FINDRISC questionnaire of the 19,951 screened respondents, the employees eligible for the program and those who agreed to participate in the program are presented in Table 2. Half of the respondents were men (*n* = 9,891; 49.6%), mean age was 38.0 ± 13.5 years and 37.0% had a BMI ≥ 25 kg/m^2^. Their mean FINDRISC score was 5.9 ± 4.4 with 802 (4.0%) participants presenting a 10-year risk of type 2 diabetes >33% (score ≥ 15). As a comparison, this high risk was recorded in 554 (15.5%) respondents >45 years with classic risk(s) factor(s) for type 2 diabetes. The prevalence of diabetes at 10 years was estimated at 4.4% overall: 5.6% in individuals ≥30 years old, 7.0% in those >45 years old, and 11.7% in those >45 years having at least one of the three main risk factors for diabetes. Relative to 1,348 eligible participants taken into account for per-protocol analysis, 1,071 (74.2%), 951 (70.5%), 834 (61.9%), 732 (54.3%), 681 (50.5%), 633 (47.0%), and 581 (43.1%) participated in the first, second, third, fourth, fifth, sixth, and seventh interviews, respectively. Among the 581 participants having completed the 7 interviews of the program, 216 were men (37.2%), the mean age was 48.6 ± 9.5 years and their mean FINDRISC score was 14.8 ± 2.5. They were slightly older, more corpulent and also had a higher FINDRISC score (14.8 ± 2.5 vs. 14.4 ± 2.4) comparatively to all eligible participants (Table 2).

**Table 2:** Characteristics of the study participants according to the different stages of the study

		*N* = 19,951 screened employees		*N* = 2,018 eligible employees		*N* = 1,348 Employees who agreed to participate		*N* = 581 participants completing the program	
		*N* (%)	Mean ± SD	*N* (%)	Mean ± SD	*N* (%)	Mean ± SD	*N* (%)	Mean ± SD
Sex	Male	9,891 (49.6)		771 (38.2)		489 (36.3)		216 (37.2)	
	Female	10,060 (50.4)		1,247 (61.8)		859 (63.7)		365 (62.8)	
Work time	Full-time	15,770 (79.0)		1,461 (72.4)		972 (72.1)		407 (70.1)	
	Part-time	4,181 (21.0)		557 (27.6)		376 (27.9)		174 (29.9)	
Work type	Sales	9,527 (47.8)		1,228 (60.9)		850 (63.1)		363 (62.5)	
	Production	10,424 (52.2)		790 (39.1)		498 (36.9)		218 (37.5)	
Age (years)			38.0 ± 13.5		47.1 ± 10.8		47.1 ± 10.5		48.6 ± 9.5
Age categories (years)	<35	8,689 (43.6)		296 (14.7)		190 (14.1)		61 (10.5)	
	35–44	3,676 (18.4)		373 (18.5)		257 (19.1)		100 (17.2)	
	45–54	4,959 (24.9)		742 (36.8)		515 (38.2)		238 (41.0)	
	55–64	2,542 (12.7)		589 (29.2)		377 (28.0)		177 (30.5)	
	≥65	85 (0.4)		18 (0.9)		9 (0.7)		5 (0.9)	
BMI (kg/m^2^)			24.3 ± 4.5		30.7 ± 4.4		30.7 ± 4.5		31.0 ± 4.4
BMI categories (kg/m^2^)^a^	Normal	12,576 (63.0)		66 (3.3)		47 (3.5)		18 (3.1)	
	Overweight	5,230 (26.2)		870 (43.1)		610 (45.3)		251 (43.2)	
	Obesity	2,145 (10.8)		1,082 (53.6)		691 (51.3)		312 (53.7)	
WC categories (cm)^b^	Normal	10,611 (53.2)		31 (1.5)		23 (1.7)		10 (1.7)	
	High	4,741 (23.8)		401 (19.9)		255 (18.9)		88 (15.1)	
	Very high	4,599 (23.1)		1,586 (78.6)		1,070 (79.4)		483 (83.1)	
Daily physical activity (yes)		14,815 (74.3)		1,038 (51.4)		672 (49.9)		297 (51.1)	
Daily consumption of fruits/vegetables (yes)		1,4506 (72.7)		1,356 (67.2)		900 (66.8)		412 (70.9)	
History of antihypertensive drug treatment (yes)		1,748 (8.8)		1,338 (66.3		881 (65.4)		369 (63.5)	
History of high blood glucose (yes)		872 (4.4)		1,566 (77.6)		1,007 (74.7)		434 (74.7)	
Family history of diabetes	1st degree relative	3,900 (19.5)		1,327 (65.8)		911 (67.6)		391 (67.3)	
	2nd degree relative	2,952 (14.8)		281 (13.9)		190 (14.1)		73 (12.6)	
	No	13,099 (65.7)		410 (20.3)		247 (18.3)		117 (20.1)	
FINDRISC score			5.9 ± 4.4		14.4 ± 2.4		14.6 ± 2.4		14.8 ± 2.5
FINDRISC categories	Very low	11,854 (59.4)							
	Low	5,784 (29.0)							
	Moderate	1,511 (7.6)		1,217 (60.3)		752 (55.8)		321 (55.2)	
	High or very high	802 (4.0)		801 (39.7)		596 (44.2)		260 (44.8)	

^a^BMI categories: normal <25 kg/m^2^; overweight 25–30 kg/m^2^; obesity ≥ 30 kg/m^2^

^b^WC (waist circumference) categories for men: normal <94 cm; High = 94–102; very high >102—for women: normal <80 cm; high = 80–88; very high >88

There was a statistically significant improvement in knowledge and habits linked to the intervention with an increase in observed scores between the first and the sixth interviews (Table 3), whether in mITT (*n* = 699/1,527 participants accepting to participate in the coaching program whatever the BMI) or PP (*n* = 630/1,348 participants eligible for the program regarding BMI) analyses (*P* < 0.0001). Similar significant trends were observed for health-related quality of life (mITT and PP analyses) and physical activities (in the mITT analysis only). These data showed an additional improvement in dietary knowledge and habits three months after the coaching program (Table 3), whether the analysis was performed in mITT (*n* = 647/1,527, *P* < 0.0001) or PP (581/1,348, *P* < 0.001). Inversely, physical activity, and quality of life scores remained similar.

**Table 3:** Effect and impact of the telephone coaching program relative to dietary knowledge and behavior, physical activity, and quality of life

	1st coaching telephone session	6th coaching telephone session	Difference between the 1^st^ and 6^th^ telephone interviews^a^	*P*^b^	Remote interview (3 months after last coaching telephone session)	Difference between the 6th and the last telephone interviews^a^	*P*^b^
Modified intention to treat analysis							
Number of participants	699	699			647		
Dietary knowledge/behavior score (1–8) median (Q1;Q3)	5 (4;6)	7 (6;8)	*2 (1;3)*	*<0.0001*	7 (6;8)	*0 (0;1)*	*0.0001*
Physical activity was measured by the GPAQ median (Q1;Q3)	3 (2;3)	3 (2;3)	*0 (0;0)*	*0.04*	3 (2;3)	0 (0;0)	0.62
QOL (SF-12,17 to 55) median (Q1;Q3)	45 (40;48)	46 (43;49)	*2 (−1;5)*	*<0.0001*	47 (43;49)	1 (−2;3)	0.12
Per-protocol analysis							
Number of participants	630	630			581		
Dietary knowledge/behavior score (1–8) median (Q1;Q3)	5 (4;6)	7 (6;8)	*2 (1;3)*	*<0.0001*	7 (6;8)	*0 (0;1)*	*0.0002*
Physical activity was measured by the GPAQ median (Q1;Q3)	3 (2;3)	3 (2;3)	0 (0*;*0)	0.07	3 (2;3)	0 (0*;*0)	0.89
QOL (SF-12) median (Q1;Q3)	45 (40;47)	46 (43;49)	*2 (−1;5)*	*<0.0001*	47 (43;49)	0 (−2;3)	0.27

^a^Difference = {score at 6th (or 7th) interview—score at 1st (or 6th) interview} calculated for each participant and median = median of all individual differences (italic characters show statistically significant differences)

Q1: 1st quartile; Q3: 3rd quartile

^b^Wilcoxon signed rank test on paired samples

Stages of change according to the Prochaska and DiClemente classification were evaluated in 653 participants. The proportion of participants considered at the “pre-contemplation”, “contemplation”, “preparation”, and “action” stages increased from 38.1% (*n* = 249), 54.5% (*n* = 356), 5.1% (*n* = 33) and 2.3% (*n* = 15) to 11.0% (*n* = 72), 17.0% (*n* = 111), 10.7% (*n* = 70), and 61.3% (*n* = 400) between the first and the seventh interviews, respectively. This favorable evolution was statistically significant (*P* < 0.0001).

## Discussion

This study, conducted on a large sample of employees from the French bakery/pastry sector, led to the identification of 4% of individuals at a high risk of type 2 diabetes. For employees from moderate to very high risk, the implementation of a health-coaching program via telephone calls appears operational. This program was associated with an improvement in knowledge and skills for dietary and physical activities, as well as quality of life. In addition, a favorable persistent increase in dietary knowledge and behavior at least 3 months after completion of the program was observed.

Our results regarding FINDRISC score in a working population are relatively similar to data from other European populations, whereas lower than that reported in the United States [29, 30]. For example, Vandersmissen and colleagues [21] studied data from 275 Belgian workers undergoing a voluntary health check and found a mean FINDRISC score slightly higher than that recorded herein (6.8 ± 4.7, with 5.5% of individuals at high or very high risk). To our knowledge, the present study is the first to estimate the 10-year risk of developing type 2 diabetes in a large sample of the French active population. A population-based study conducted in 67 pharmacies in northeast France from 2007 to 2010 compared the capillary blood glucose measurement and FINDRISC questionnaire in 1907 individuals over 45 years of age having at least one of the main risk factors for type 2 diabetes. The study authors reported that 16.6% of the participants had a FINDRISC ≥ 15, which is close to the 15.5% found in the present study [31]. As well, recent data concerning >99% of beneficiaries of a health insurance scheme in France showed an estimated prevalence of pharmacologically treated diabetes, which reached 5% in 2015 [32]. This rate is similar to this estimated herein in individuals ≥30 years old. Hence, it could be argued that the risk of type 2 diabetes in French bakery/pastry employees likely does not differ from the overall French adult population.

Progression of prediabetes to type 2 diabetes may be prevented through favorable lifestyle interventions [18]. However, initiating and maintaining healthy lifestyle changes remain a genuine challenge [33]. Health coaching through telephone interviews is one of the emerging tools that have been shown to improve favorable health behaviors in patients suffering from chronic diseases such type 2 diabetes, as well as for prediabetes [34, 35]. With regard to studies performed in the workplace, Wilson et al. [36] published selected data from a randomized controlled trial conducted in 418 city/county employees in order to analyze the impact of the “Fuel Your Life” program. Three interventions were used: small group coaching, self-study and telephone coaching, the latter representing the best strategy with 28.3% of the participants of this group losing 5% or more of their body weight [36]. As our strategy, a few authors have proposed to condition a coaching-type intervention to a risk estimation by the FINDRISC score, which was also positively used as a criterion of effectiveness [37]. Finally, the telephone coaching proposed in the present study shows encouraging results in terms of favorable dietary behaviors and commitment toward an enhanced prevention of type 2 diabetes. Telephone coaching is one of the available technology-assisted interventions for diabetes prevention, even if the ideal combination of these technologies still necessitates further assessment and impact studies, including its cost-effectiveness [38–40]. For example, such a positive and lasting impact is not found in all studies despite enhanced physical activity [41].

The substantial number of included individuals herein for screening and intervention constitutes one of the strengths of the present study. Indeed, other published data including such high number of individuals from the professional sector remain rare [29]. However, our results should be interpreted with caution. For the screening survey, we cannot affirm that all those who were not contacted by telephone or did not wish to participate did not differ from respondents. Moreover, answers to all items of the FINDRISC questionnaire were self-reported, such that we cannot exclude errors in certain responses. The choice of the FINDRISC score can also be debated. Indeed, other non-biological tools are also available. For example, the German Diabetes Risk Score was derived from the European Prospective Investigation into Cancer and Nutrition (EPIC)-Potsdam Study and includes information on age, waist circumference, height, history of hypertension, physical activity, and consumption of alcohol, coffee, whole grains, and red meat [42]. The American Diabetes Association type 2 diabetes risk test is also widely used and includes seven questions for which a user can score up to 11 points with the threshold for people at risk being at 5 points [10]. Other scores are well published, including the Canadian diabetes risk questionnaire (CANRISK), the Australian type 2 diabetes risk assessment tool (AUSDRISK), and the QDiabetes risk model built from a large cohort of patients in England and Wales [43–45]. All of these risk scores show overall good discriminatory ability in populations for whom they were developed [46]. However, discriminatory performance is more heterogeneous and generally weaker in external populations [47]. Other limitations of this study include the short time span (3 months later) used to evaluate the impact of the coaching program and the lack of a control group. Finally, a short self-constructed questionnaire was used for dietary knowledge and behavior (four questions per domain), which was designed for this specific study and not validated by adapted studies [48]. Although our data suggest that targeting both nutritional knowledge and motivation can together mobilize skills and likely favor type 2 diabetes prevention, it is nonetheless difficult in this particular instance to distinguish the effects between acquiring knowledge and boosting motivation per se, both being also related to the quality of the relationship [49]. Another point is the acceptability of such a program: the number of individuals who ultimately accepted and participated to all interviews of the coaching program could be considered as low (approximately one-third) compared with the number of individuals at high risk. However, prevention programs aimed at reducing this risk can have a significant and large impact if they are implemented on a large scale [50].

## Conclusion

Although the observed data remain to be confirmed, this study demonstrated that employees from the French craft bakery/pastry sector have a similar risk of type 2 diabetes than other general European populations, including France. The combination of risk screening using the FINDRISC score and the 6-month telephone health-coaching program conducted in a professional environment furthermore appears effective, showing encouraging results on both motivation and favorable behaviors, which could help prevent type 2 diabetes.
